# Towards a theory-informed framework of child-centred care in paediatric healthcare: a systematic scoping review

**DOI:** 10.3389/fped.2026.1900023

**Published:** 2026-09-04

**Authors:** Patricia DeCosta, Dan Grabowski, Tue Helms Andersen, Imelda Coyne

**Affiliations:** 1 Department of Prevention, Health Promotion and Community Care, Copenhagen University Hospital – Steno Diabetes Center Copenhagen, Herlev, Denmark; 2 Department of Health Services Research, University of Southern Denmark, Odense, Denmark; 3 Department of Education, Copenhagen University Hospital – Steno Diabetes Center Copenhagen, Herlev, Denmark; 4 School of Nursing and Midwifery, Trinity College Dublin, Dublin, Ireland

**Keywords:** child participation, child-centred care, paediatric healthcare, scoping review, theoretical framework

## Abstract

**Introduction:**

Child-centred care is increasingly advocated in paediatric healthcare, yet its theoretical foundations remain fragmented and poorly defined. This review aimed to identify and synthesize theoretical perspectives applied to child-centred care in healthcare and to develop a theory-informed framework that clarifies its theoretical foundations and operationalisation.

**Methods:**

The review followed the JBI methodology for systematic scoping reviews and was conducted and reported in accordance with the PRISMA Extension for Scoping Reviews (PRISMA-ScR). A comprehensive search of MEDLINE, Embase, and CINAHL was conducted for studies published between 1946 and August 2024. Peer-reviewed studies involving children, parents, or healthcare professionals that applied theoretical perspectives to child-centred care were included. Screening and data extraction were conducted independently by multiple reviewers. Data were synthesized in three steps: (1) grouping studies by theoretical framework, (2) analysing shared elements across six theories, and (3) integrating themes to construct a framework and identify core domains.

**Results:**

Thirteen studies were included, identifying six theoretical perspectives underpinning child-centred care. Synthesis of these perspectives resulted in a theory-informed framework comprising three interrelated domains: (1) the competent child, (2) social context and ecologies, and (3) development as a dynamic process. Together, these domains conceptualise child-centred care as a relational, contextual, and developmental process that supports children's participation, agency, and well-being.

**Conclusion:**

This review advances conceptual clarity by integrating six theoretical perspectives into a theory-informed framework of child-centred care. The framework positions child-centred care as a dynamic, relational, and developmental process linking children's agency, social context, and development. The framework provides a tentative, structured foundation for guiding practice, education, and research. Further research is required to test and refine the framework across diverse cultural, geographic and healthcare contexts.

## 1 Introduction

Our understanding of the psychological care needs of children receiving healthcare has evolved over time and come to increasingly prioritize the family and the child within the care framework (1). The United Nations Convention on the Rights of the Child (1989) reinforced this shift by affirming children's rights to express their views and participate in decisions affecting their care. This perspective positions children as active participants, rather than passive recipients, aligning with global movements towards child- and family-centred care (2).

Empirical research has consistently shown that many children and young people wish to participate actively in their healthcare through honest, developmentally appropriate communication and opportunities to express their views and preferences (3–6). However, meaningful participation remains inconsistent in practice, and many children report feeling excluded from consultations or insufficiently heard by healthcare professionals (3, 4, 7). Children's involvement often depends on individual clinicians' attitudes and practices rather than being systematically embedded within healthcare delivery (4). The persistent gap between children's expressed need for meaningful participation and its inconsistent implementation in clinical practice underscores the need for greater theoretical clarity to guide the development, implementation, and evaluation of child-centred care.

Compared to patient- and family-centred care, child-centred care (CCC) is a relatively new and evolving concept and has been inconsistently defined in the literature (8). Although family-centred care remains the dominant philosophy in paediatric healthcare, its emphasis on the family as the unit of care may inadvertently overshadow the child's own perspective if parental involvement becomes the principal focus (9). In response to this limitation, Coyne and colleagues' concept synthesis defined CCC as a distinct approach that places the child at the centre of all healthcare interactions, recognising children as capable, active participants in their care whose voices, rights, needs, preferences should shape communication, participation, and decision-making processes, while maintaining strong partnerships with families (2) As children are the recipients of healthcare and the individuals directly experiencing illness and hospitalisation, their needs and preferences must remain central to care delivery (2).

However, a recent scoping review (10) found no clear consensus on the definition of CCC in the literature, but did identify four core elements: agency, participation, decision-making, and communication. The review also noted a lack of clarity on how these elements could be meaningfully put into practice. They concluded that CCC remains an emerging and poorly defined concept, with limited evidence of its impact or benefits (10).

While assessing effectiveness is important, evaluating a concept that lacks conceptual clarity risks producing flawed conclusions. A more immediate priority is to examine the theoretical perspectives on CCC to provide the clarity and precision needed to guide future research and practice. We define a theoretical perspective as a broader framework or lens through which to approach an area of study. It represents a particular viewpoint or set of assumptions about how to analyse and understand the world.

We define theory as a structured and empirically supported framework of interrelated constructs (concepts), definitions, and propositions that provides a systematic and purposeful view of phenomena (11). Theories offer guidance, explanation, and prediction, thus helping to deepen our understanding of a subject, such as how CCC may benefit children and through what mechanisms. As the integration of psychological and social needs in paediatric healthcare is complex and continually evolving, theories offer a lens through which to interpret results and connect new findings with existing knowledge. This contextualization is essential for making meaningful contributions to the evidence and extending our understanding of the field (12). Theoretical knowledge also ensures that research aligns with established principles, producing findings that contribute to broader frameworks (13). When exploring emerging concepts such as CCC, theory can uncover relationships and insights that may not be immediately apparent, thus fostering creativity and innovation.

Given the conceptual ambiguity, a scoping review is warranted to systematically examine and synthesize the theoretical foundations of CCC in healthcare. Understanding how the concept has been defined or theorized over time provides important context for its evolution and changing meaning, placing it within a broader intellectual tradition and revealing how different scholars have approached it. Theoretical synthesis, which integrates propositions from multiple theories into a cohesive framework, can enhance coverage, strengthen validity, and identify points of convergence (14). Furthermore, reviewing theoretical perspectives applied to CCC can expose gaps, identify areas of consensus, and highlight where more refined or updated definitions are needed. This will deepen our understanding of the concept and inform future research and practice in CCC.

### 1.1 Aim and objectives

Given CCC relevance as a healthcare approach, this scoping review aims to identify and synthesize literature that applies theory or theoretical perspectives to elucidate CCC and its operational aspects in healthcare settings. To achieve this aim, the review was guided by the following research questions:

- What theories or theoretical perspectives have been employed in studies investigating CCC?
- What are the fundamental elements considered within the CCC approach?
- What insights can be gained from theory regarding CCC as an approach to caring for children and young people?

## 2 Method

This scoping review has been conducted in accordance with the JBI methodology for scoping reviews (15) and reported in accordance with the PRISMA-ScR (PRISMA extension for ScopingReviews) (16).

### 2.1 Protocol and registration

A scoping review protocol was developed in accordance with Peters, Godfrey (17) “Best practice guidance and reporting items for the development of scoping review protocols”. The final review protocol was registered on the Open Science Framework in January 2025: (see title page)

In preparation for this scoping review, an initial literature search was conducted in October 2024. No review examining the theoretical foundation for CCC was identified.

### 2.2 Eligibility criteria

Participants: Studies involving at least one of the following populations were eligible: (1) children and young people aged 0–18 years, with or without an illness/diagnosis, receiving some form of healthcare; (2) parents or caregivers of children and young people (0–18 years) receiving healthcare; or (3) healthcare professionals (HCPs) providing healthcare to children and young people.

Concept: The present scoping review included articles that employed theories or theoretical perspectives as frameworks for investigating, understanding, or underpinning child-centred care or patient-centred care applied to children.

Context: The review included literature on CCC in healthcare settings, such as hospitals (including surgery, diagnostics, specialized treatments, and emergency care), outpatient clinics, primary care, and healthcare services provided in schools or the home.

To be included in the scoping review, articles must have been published in peer-reviewed journals. This included primary studies (empirical and theoretical), systematic reviews and discussion papers. Quantitative, qualitative and mixed-method studies were included. Articles were included if they were written in English, French, German, Danish, Norwegian, or Swedish, as these corresponded with the language abilities in our research team.

Articles were excluded if they did not address CCC using a theoretical framework, or theoretical perspective, conducted outside relevant healthcare settings, were not peer-reviewed, did not meet study type criteria, or were published in other languages. Studies that referred to CCC solely in the context of transitioning from paediatric to adult care settings were excluded from the review.

### 2.3 Information sources

A literature search from inception to 3 September 2024, was conducted in MEDLINE, Embase and CINAHL. The search was updated in all databases on 22 September 2025.

The databases were selected to provide comprehensive coverage across a diverse range of literature, encompassing life sciences and biomedical disciplines as well as nursing and healthcare-related subjects. The final search results were exported into the software program EPPI-Reviewer 6 (18) and duplicates were removed by an information specialist (XX).

### 2.4 Search

The search string (see Appendix) contained both Medical Subject Heading (e.g., MeSH) terms and free text words describing the relevant concepts. The search strategy was developed with an experienced information specialist (XX) who also conducted the search. It was developed in MEDLINE and subsequently translated to the other databases. The search was evaluated by testing if key literature, already known to the authors, and identified though previous reviews on CCC was retrieved (2, 10). It successfully retrieved all the relevant literature in question.

Citation tracking was performed by retrieving all references (backward) and citing articles (forward) of the included articles, with the software tool citationchaser (19).

### 2.5 Selection of sources of evidence

To ensure consistency among reviewers, the team jointly screened the first 20 records based on titles and abstracts, discussed the results, and refined the inclusion and exclusion criteria accordingly. Following this, sources were initially screened by title and abstract. Retained sources were then assessed in full text. Reasons for exclusion at the full-text stage were documented.

The screening and selection of sources of evidence were conducted independently by the first author (XX) and a second reviewer. Both reviewers assessed all records against the predefined eligibility criteria at the title/abstract and full-text stages. Any discrepancies between reviewers were resolved through discussion to reach consensus. If consensus could not be achieved, a third (XX) and, if necessary, a fourth reviewer (XX) were consulted to make a final decision. In total, five articles were reviewed by all four reviewers in order to reach consensus.

The included articles' references and citing articles were also screened to identify additional relevant sources. The overall selection process was documented and is presented in a flow diagram.

Data were managed in EPPI-Reviewer throughout all stages of the review process, including reference management, screening, and coding (title/abstract screening and full-text review). The software enabled multiple reviewers to work simultaneously and track progress.

### 2.6 Data charting process

A structured data charting form was developed specifically for the present review to extract key information from the included sources. Consistent with our review questions, we extracted information on the theories or theoretical perspectives that had been employed in each included study, as well as information on the fundamental elements of CCC. The chart included the following fields: source of evidence (citation/place of study), aim, participant characteristics, definition of CCC, method (theoretical/empirical), theories or theoretical perspectives employed, and fundamental elements of CCC.

The data chart was pilot tested on a small sample of included studies, and minor adjustments were made based on the team's feedback to improve clarity and consistency. The charting process was conducted by the first author (XX), who extracted data from all included sources. Charted data were then validated by (XX) and (XX) to ensure accuracy and completeness. Any uncertainties or disagreements were resolved through group discussion until consensus was reached. The charting process was iterative, allowing for refinement of the form as needed to capture emerging concepts or clarify core theoretical elements.

### 2.7 Critical appraisal of individual sources of evidence

Consistent with the objectives of a scoping review, formal critical appraisal was not undertaken. We focused on describing the level of use of theory within each study, as this was considered more informative for understanding how theory guided design, analysis, or argumentation (20). To capture this, we applied a three-level rating:

Level 1—Minimal/Implicit: At this level, theory is primarily used to provide background or contextual framing. Its application to the analysis or interpretation of findings is unclear or not explicitly demonstrated.
Level 2—Moderate/Conceptual: Here, theory guides the conceptual framing of the study or informs part of the analysis. Integration of theory is partial, and connections between theoretical constructs and findings or arguments are present but not consistently applied.
Level 3—Strong/Integrated: Theory is explicitly applied in analysis, whether guiding the study design or applied after data collection. Theoretical concepts are operationalized and systematically linked to findings or discussion points.

### 2.8 Use of artificial intelligence tools

ChatGPT (OpenAI) was used to assist in the articulation of the three theoretical levels described above. The tool supported phrasing and refinement of descriptions based on author-provided content and guidance.

## 3 Results

**Figure 1:**
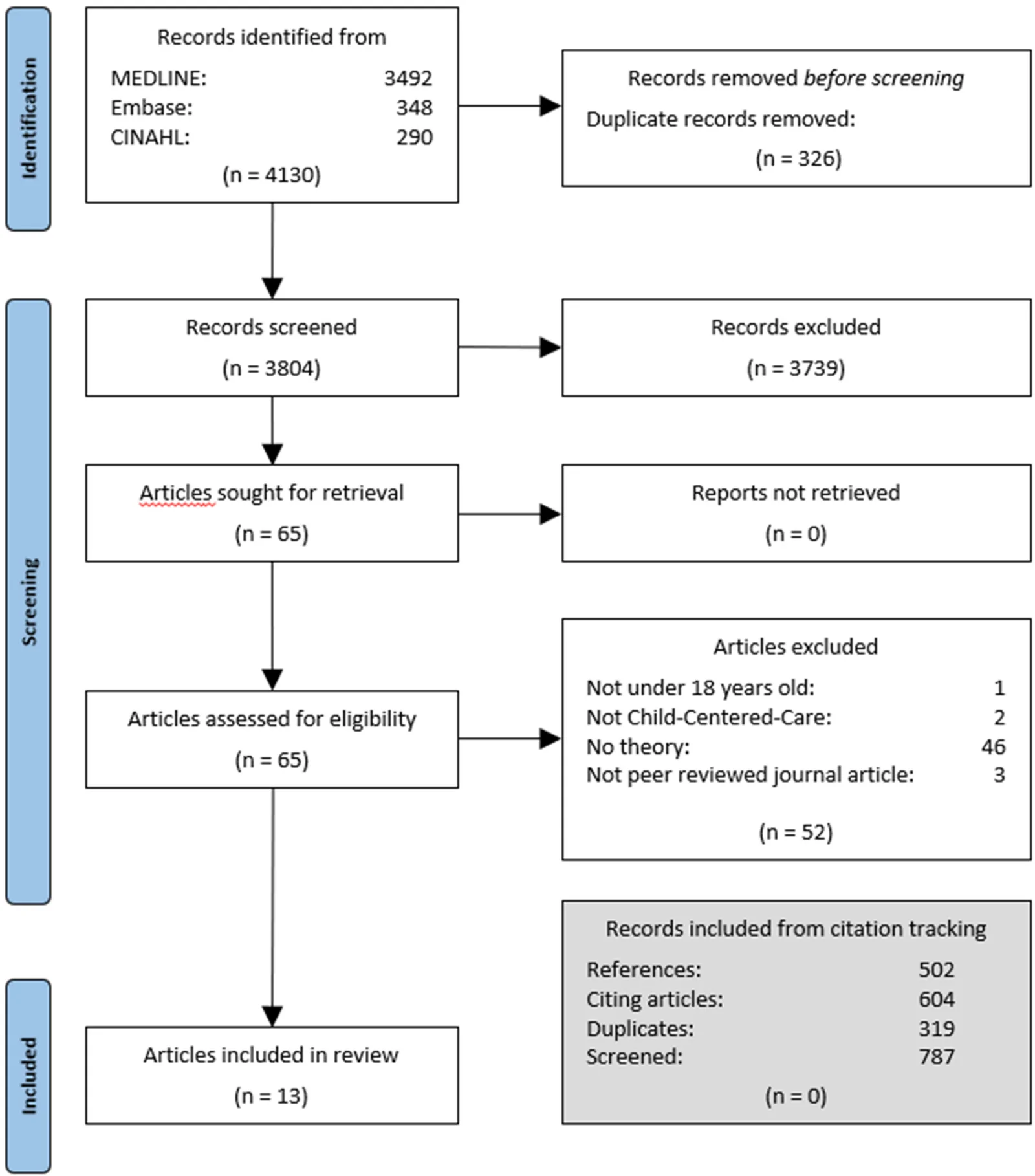
Flow diagram of the selection process.

The database search yielded 4,130 records. After duplicates were removed, a total of 3,804 records were screened on title and abstract. Based on the title and the abstract, 3,739 records were excluded, with 65 full text articles retrieved and assessed for eligibility on full text. Of these, 52 were excluded for the following reasons: 1 did not include a population of people under the age of 18, 2 did not focus on child-centred care (or similar), 46 did not use a theory or theoretical perspective to analyse data, and 3 were conference abstracts, and not published or peer reviewed literature. The remaining 13 studies were considered eligible for this review. The citation tracking retrieved 1,106 records (502 references and 604 citing articles). After removing 319 duplicates, 787 were screened by title and abstract and 9 articles were screened full text. None were excluded leaving a final of 13 included articles. For more details see Figure 1.

### 3.1 Overview of included studies

Empirical studies were conducted in the UK (21, 22), Australia (23, 24), Denmark (25, 26) and Sweden (27). The theoretical discussion (and review) papers were authored by people from NZ (28), Australia (28, 29), Ireland (9, 30), Sweden (9, 30), Denmark (31), the UK (32) and Malta (32). The studies were published between 2008 and 2022.

Eight studies were based on empirical data (21–27, 31), and five were theoretical discussion papers (9, 28, 30, 32, 33). Eight studies were rated as Level 3, indicating strong theoretical integration, where theory was explicitly applied to guide study design, and/or analysis, and interpretation (9, 21, 25–27, 30, 32, 33). Five articles were rated as Level 2, reflecting moderate use of theory that informed conceptual framing but lacked consistent integration into analysis or findings (22–24, 28, 31). Importantly, each theoretical view identified was represented by at least one article rated at Level 3, demonstrating that each theory provided a strong and coherent rationale for understanding CCC.

Six theories were identified in the present review. These were Bronfenbrenner's **Ecological Model** (21–24, 27, 28), **The New Sociology of Childhood and the Child's Perspective** (9, 30), Vygotsky's Zone of **Proximal Development** (25), **Resilience Theory** (31, 33), **Trust Theory** (26), and The **SECI Model and the Concept of Ba** (32).

Bronfenbrenner's Ecological Model emerged as an overarching or grand theory. It offers a broad lens, through which the child is viewed as situated within, and shaped by, multiple interconnected systems such as family, school, and society. This model provides the ecological foundation within which all other theories can be positioned, reinforcing the centrality of the child in a dynamic, multi-layered environment.

The Child's Perspective and the New Sociology of Childhood were identified as the philosophical grounding of CCC. These perspectives emphasize children's rights, agency, and voices, framing children as active social participants and meaning-makers. They offer the ethical and conceptual justification for child-centred approaches.

The remaining four theories: Vygotsky's Zone of Proximal Development, Resilience Theory, Theory of Trust, and the SECI Model, aligned closely with Bronfenbrenner's Ecological Model, as well as with the philosophical grounding of the Child's Perspective, which emphasizes agency and voice. They explore the mechanisms, interactions, and conditions that support meaningful engagement with children, and they provide practical insights into fostering development, trust, resilience, and relational knowledge in real-world settings. Together, the six theoretical perspectives operationalize the principles of CCC, demonstrating how philosophical commitments to children's agency and contextual embeddedness can be translated into responsive, relational, and participatory practices.

### 3.2 Synthesis of results

Our synthesis followed a three-step process. In Step 1, we extracted and summarized key information from each study, including its aims, theoretical perspective, and findings, and then grouped the studies by their primary theoretical frameworks (Table 1). In Step 2, we conducted a thematic synthesis across the six theories to identify shared perspectives and conceptual alignments, thereby outlining the theoretically grounded elements of CCC (Table 2). In Step 3, we refined and integrated these themes to generate a framework that highlights how the theories collectively inform both the philosophical and practical foundations of CCC.

**Table 1:** Data extraction tool.

Source of evidence (citation)/place of study	Aim	Participant’s characteristics	Definition of child-centred care	Method/level of theoretical use	Theories or theoretical perspectives employed	Key theoretical findings/fundamental elements of child-centred care
Ecological model						
(21) Brigden, A., Shaw, A., Barnes, R., Anderson, E., & Crawley, E. (2020). The child's got a complete circle around him”. The care of younger children (5–11 years) with CFS/ME. A qualitative study comparing families’, teachers’ and clinicians’ perspectives. Health & Social Care in the Community, 28 (6), 2179–2189 (UK)	To examine how the care of younger children (aged 5–11 years) with Chronic Fatigue Syndrome/Myalgic Encephalomyelitis (CFS/ME) is shared across home, education, and health settings. The study seeks to understand the barriers to integrated care and generate recommendations for improving the integration of care across these settings.	8 children with CFS/ME (5–11 years). 14 parents. 11 school staff. 9 clinicians.	CCC was not explicitly defined but emerged through the analysis in terms of identification of the child's “goals”, “having their voice in the room”, giving children “ownership”, and encouraging communication.	Empirical study, theoretical framework. Level 3—Strong/Integrated: *Theory is systematically applied to guide the study design, structure the analysis, and interpret findings across multiple levels of influence.*	Socio-ecological theory (Bronfenbrenner, 1979; Richard, Gauvin, & Raine, 2011).	A socio-ecological perspective, which considers the child within wider systems, is beneficial for understanding and managing their care. The most effective support for children with chronic health conditions involves targeting these surrounding systems. This includes parents, teachers, and healthcare providers, as well as addressing education and health policies that can facilitate shared-care approaches.
(22) Clarke, S. (2022). An exploration of the child's experience of staying in hospital from the perspectives of children and children's nurses using child-centered methodology. Comprehensive Child and Adolescent Nursing, 45 (1), 105–118. (UK)	To explore the views and feelings of children admitted to hospital. Secondly, to explore the views and experiences of registered children's nurses and identify any differences and similarities in how the child's hospital stay is perceived by the child and the registered children's nurse.	18 children (6–12 years) 8 children's nurses	CCC was not defined. They looked at the views, feelings and needs of children in hospitals wards, using a child-centred method.	Empirical study, theoretical framework. Level 2—Moderate/Conceptual: *Theory is used to frame the research conceptually, but application in analysis and linkage to findings is limited and inconsistent.*	(85)	Hospitalized children value being listened to, having choices, access to play, and the presence of their parents. They preferred child-friendly environments with personalized spaces and needed their fears and concerns addressed to promote emotional well-being. Children with frequent or extended hospital stays often formed trusting relationships with nurses, integrating them into their microsystem.
(28) Ford, K., Dickinson, A., Water, T., Campbell, S., Bray, L., & Carter, B. (2018). Child centred care: challenging assumptions and repositioning children and young people. Journal of Pediatric nursing, 43, e39–e43 (New Zealand, Australia, UK)	To explore the concept of CCC and the differences and similarities in relation to FCC. And its potential theoretical alignment with an ecological approach to healthcare.	NA	CCC acknowledges the agency of children and young people, viewing them as social actors and advocates for their right to participate in healthcare decisions. Key principles include a holistic view of the child beyond their illness; focus on the overall experience of the child and family; recognition of children, young people, and parents as care partners; advocacy for services tailored to their needs; support for transitions to adult services; and using the child's interests as the starting point for care planning (86, 87).	Theoretical Level 2—Moderate/Conceptual: *Theory is clearly articulated and used to frame the discussion, but its integration remains conceptual, with arguments informed by theoretical models rather than systematically structured or operationalized through them.*	Bronfenbrenner's Ecological Model (Bronfenbrenner, 1986, Bronfenbrenner, 1999)	In healthcare, the child, parents, and professionals are interconnected with one another, as well as with their broader environment. This perspective aligns with an ecological framework of CCC, which emphasizes the child's central role while recognizing the significance of their family and acknowledging that “a child is a child through their family”.
(23) Foster, M., Quaye, A. A., Whitehead, L., & Hallström, I. K. (2022). Children's voices on their participation and best interests during a hospital stay in Australia. Journal of Pediatric Nursing, 63, 64–71. (Australia)	To explore school-aged children's experiences concerning their best interests and participation in care during a hospital admission.	9 children/YP (5–15 years) and their parents	The child is recognized as a capable and active participant in their own right. CCC is planned with the child's self-reported views and preferences in mind, guided by adults who consider the child's competence within a family and community context (9, 88). A child's ability, choice, and opportunity to engage in shared decision-making is an ongoing, evolving process, shaped by what the child identifies as being in their best interest (8, 9, 89).	Empirical study, theoretical framework. Level 2—Moderate/Conceptual: *Theory is used to frame the research conceptually, but its integration into the analysis and discussion is partial, with limited and inconsistent connections to the findings.*	Bronfenbrener's bioeological model of human development (34)	Children were able to express their best interests and suggest ways to enhance their participation in hospital care. Their interactions with parents, HCPs, and the environment reflected the interconnected systems in Bronfenbrenner's bioecological model. Documenting children's self-reported experiences is essential to guiding CCC. Supportive relationships and environments are key to promoting their participation and well-being. Co-designing age-appropriate settings with children can further support these goals.
(24) Nicholl, A., Evelegh, K., Deering, K. E., Russell, K., Lawrence, D., Lyons-Wall, P., & O'Sullivan, T. A. (2020). Using a Respectful Approach to Child-centred Healthcare (ReACH) in a paediatric clinical trial: A feasibility study. PLoS One, 15 (11), e0241764. (Australia)	To determine the acceptability and effectiveness of the Respectful Approach to Child-centred Healthcare (ReACH) in obtaining compliance with healthcare assessments in a clinical trial.	49 children (4–6-year-olds) and their parents.	The Respectful Approach to Child-centred Healthcare (ReACH) is based on a child-centred philosophy and emphasizes using authentic communication, acknowledging emotions, and encouraging active participation and play. The approach advocates for providing appropriate information and education to both children and caregivers, avoiding distractions or incentives, and ensuring that healthcare workers recognize and respect children's verbal and non-verbal dissent.	Empirical study, theoretical framework. Level 2—Moderate/Conceptual: *Theory informs the development of the ReACH model and supports the conceptual framing, but its application in the results and discussion is limited, with minimal linkage between theoretical concepts and findings.*	Bronfenbrenner's ecological model of child development (56, 85)	Healthcare and research institutions should have an appropriate child-centred ethical model that upholds children's rights. A holistic child-centred approach places the child at the centre, focusing on building mutual trust, maintaining honest communication, using sensitive observation to understand needs, and creating a safe, supportive environment.
(27) Stålberg, A., Sandberg, A., Larsson, T., Coyne, I., & Söderbäck, M. (2018). Curious, thoughtful and affirmative—Young children's meanings of participation in healthcare situations when using an interactive communication tool. Journal of Clinical Nursing, 27 (1–2), 235–246. (Sweden)	To describe how young children demonstrate participation in healthcare situations while using an interactive communication tool jointly with HCPs.	20 children (3–5 years), attending either a primary healthcare clinic or a paediatric outpatient unit at a hospital.	CCC was not defined.	Empirical study, theoretical framework. Level 3—Strong/Integrated: *Theory is applied throughout the study and systematically linked to children's observed behaviours and meanings of participation.*	Bronfenbrenner's ecological model of child development (85) children as social actors and competent co-constructors in social relations (37, 38) The child's perspective (40) Guided participation (90, 91)	Child agency, guided participation, and adopting the child's perspective are core elements of a CCC approach. An interactive tool facilitated shared understanding and supported children's learning and participation. Children expressed cues of participation (curious, thoughtful, and affirmative) demonstrating active engagement. These findings portray the child as a competent social actor and reinforce the need to genuinely listen to children's expressions and meanings, in line with UNCRC principles.
The child's perspective						
(9) Coyne, I., Hallström, I., & Söderbäck, M. (2016). Reframing the focus from a family-centred to a child-centred care approach for children's healthcare. Journal of Child Health Care, 20 (4), 494–502. (Ireland, Sweden)	To identify key strengths and challenges of FCC and CCC. To propose that the care of children in healthcare needs to be reframed through a child-centred lens and to describe how such a move would influence clinical practice.	NA	The child, within a family, is the central and active agent in the partnership. The child's agency is recognized, along with their needs and rights to privacy and dignity. They are involved in decisions affecting their care, based on their level of competence. The child is viewed as a social actor with their own rights. The child is included and guided by an adult, offering opportunity to increase competence. Care is planned around the child's perspective and preferences, in the context of family and community.	Position statement using theoretical concepts Level 3—Strong/Integrated: *Uses theory to systematically structure its arguments, consistently linking conceptual ideas to discussion points and practical implications in a clear and integrated way.*	The new sociology of childhood and the child's perspective (39, 40)	The child's perspective is not prominent or the focus of FCC. Therefore, care needs to be redirected towards a child-centred approach, as it encompasses the child's right to participate in all aspects of healthcare delivery in conjunction with the needs of their family. A move towards CCC is consistent with governmental policies and national reports in Europe. Adopting a child-centred approach involves recognizing and focusing on children's agency and rights, while valuing their voices, experiences, and participation.
(30) Söderbäck, M., Coyne, I., & Harder, M. (2011). The importance of including both a child perspective and the child's perspective within health care settings to provide truly child-centred care. Journal of Child Health Care, 15 (2), 99–106. (Sweden, Ireland)	To use research to illuminate and differentiate between a child perspective and child's perspectives in healthcare and in relation to providing CCC.	NA	CCC requires health professionals with expertise in child development, life conditions, and the specific circumstances of each child. They need to be knowledgeable about children's rights. Professionals should use this knowledge to apply a child perspective in any given situation while also considering the child's own viewpoint. The ability to balance these perspectives depends on the professional's skill in perceiving and understanding the child's world (40)	Theoretical Level 3—Strong/Integrated: *Theory structures the paper's core argument in a clear and systematic way, with conceptual distinctions consistently applied to critique practice and support recommendations for child-centred care.*	The child's perspective (40)	Both a child perspective and the child's perspective are essential to perceiving and engaging with children as equal human beings in child-centred healthcare settings. The approach of family-centred care should be redirected towards a CCC approach, which incorporates the child's right to participate in all aspects of healthcare delivery alongside the needs of their family.
Trust						
(26) DeCosta, P., Skinner, T. C., & Grabowski, D. (2021). The Role of Trust in the Care of Young Children with Type 1 Diabetes. Children, 8 (5).(Denmark)	To understand the role of trust in young children's psychosocial care needs after a type 1 diabetes diagnosis, from the perspectives of HCPs. Secondly, using theory, the study examines the trust-building process between young children and HCPs, aiming to identify key bases and barriers to trust in diabetes care.	20 HCPs from 11 paediatric diabetes clinics. 11 paediatric nurses, 4 paediatricians, 3 psychologists, 2 dietitians.	The article does not define CCC. They describe a need to understand and identify young children's experiences, as well as their need for support, as the foundation and prerequisite of offering CCC.	Empirical study, theoretical framework Level 3—Strong/Integrated: *Theory is explicitly applied to guide the analysis and interpretation, with conceptual elements systematically linked to findings and used to explain the trust-building process in depth.*	Theory of trust (46)	Trust is a central element in CCC. Key elements include building trust through meaningful interactions like play, establishing trust at diagnosis to reduce fear, build long-term trust with consistent HCPs, and caregivers acting as a bridge to trust. An experience of trust helps to counteract medical anxiety, ensuring effective care. Trust is a long-term investment that begins at diagnosis and continues through childhood, with caregivers playing a vital role in mediating trust between children and HCPs.
Zone of Proximal Development (ZPD)						
(25) DeCosta, P., Grabowski, D., Jespersen, L. N., & Skinner, T. C. (2022). Playful Communication and Care: Exploring Child-Centred Care of Young Children With Type 1 Diabetes Through the Framework of Zone of Proximal Development. Front. Clin. Diabetes Healthc, 2. (Denmark)	To explore current care practices for young children with type 1 diabetes and identify aspects of CCC successfully integrated into current practice, through the knowledge and practical experience of HCPs.	20 HCPs from 11 paediatric diabetes clinics. 11 paediatric nurses, 4 paediatricians, 3 psychologists, 2 dietitians.	CCC involves being responsive to children's views and preferences; eliciting the child's perspectives; adapting care to the individual child's needs; fostering relationships and providing children with the time and opportunity for participation (9)	Empirical study, theoretical framework Level 3—Strong/Integrated: *Theory is explicitly applied to guide the analysis, operationalized through the identification of CCC practices, and consistently linked to findings. Conceptual ideas are systematically used to interpret results and inform practical recommendations.*	Vygotsky's (41) theory of ZPD and the concept of Scaffolding (42)	For care to be child-centred, interactions need to be within the young child's ZPD. To achieve this, HCPs use play-based approaches as scaffolding to support children, helping them understand and participate in their care. Play serves as a developmental tool to create a safe and engaging environment, allowing children to express emotions and comprehend information. Meaningful participation involves actively engaging children in age-appropriate activities, enhancing their understanding and reducing anxiety. These elements underscore the importance of ZPD in fostering children's development and engagement in healthcare.
Resilience						
(33) Noeker, M., & Petermann, F. (2008). Resilienz: Funktionale Adaptation an widrige Umgebungsbedingungen. Zeitschrift für Psychiatrie, Psychologie und Psychotherapie, 56 (4), 255–263. (Germany)	The article provides a conceptual framework of resilience for understanding how successful coping episodes, supported by cognitive and behavioural competencies, contribute to the development of resilience; it outlines parent- and child-centred intervention strategies to foster resilient developmental processes in adverse contexts.	NA	The article does not use or define the term CCC. They refer to child-centred interventions aimed at supporting children and adolescents.	Theoretical Level 3—Strong/Integrated: *Explicitly applies theory to guide its analysis, systematically operationalizes key concepts like protective factors and moderator variables, and consistently links these to empirical findings and clinical implications.*	Resilience theory (33, 45)	Child-centred interventions foster resilience by supporting problem-solving, emotional regulation, and self-efficacy. Positive reinforcement and exposure to manageable challenges enhance coping and adaptation. The Developmental Model of Resilience highlights the dynamic interplay between children's personal competencies and their environments, positioning children as active participants in their resilience development through continuous learning and adaptation in real-life stress situations. Biological traits, temperament, and social support also shape outcomes, reinforcing the importance of individualized, context-sensitive approaches.
(31) DeCosta, P., Grabowski, D., & Skinner, T. C. (2020). The psychosocial experience and needs of children newly diagnosed with type 1 diabetes from their own perspective: a systematic and narrative review. Diabetic Medicine. (Denmark, Australia)	To understand children's psychosocial experience and identify their primary needs for support following diagnosis with type 1 diabetes.	The review included data on 134 children (3–14 years)	The article uses the terms CCC and child-centred support, but does not define these concepts.	Review/ Theoretical discussion Level 2—Moderate/Conceptual: *Theory is used to conceptually frame the discussion and guide recommendations, but it is introduced after analysis and not systematically applied to interpret findings.*	Resilience theory (43, 44)	Key concepts of resilience theory align closely with children's self-expressed support needs and offer a framework to guide clinical practice and research. Resilience theory helps to understand children's needs and support their psychosocial adjustment. At diagnosis, children needed time for emotional adjustment, supportive relationships, meaningful participation, engagement, and to feel supported without being singled out. Meeting these needs helps children navigate diagnosis by fostering autonomy, problem-solving, and social competence, thereby enhancing resilience through CCC and support that addresses their psychosocial needs.
SECI model and concept of Ba						
(32) Attard, C., Elliot, M., Grech, P., & McCormack, B. (2022). Adopting the concept of “Ba” and the “SECI” model in developing person-centered practices in child and adolescent mental health services. Frontiers in Rehabilitation Sciences, 2, 744146. (UK, Malta)	To explore how the concepts of Nonaka's SECI model and Ba can be adapted and used to develop person-centred practices within child and adolescent mental health services.	NA	The article refers to The World Health Organization definition of people-centred health services as those that put people over their disease and empower them to actively participate in their own care. It emphasizes a humane, holistic approach that consciously considers the perspectives of individuals, their families, and communities (32)	Theoretical Level 3—Strong/Integrated: *Theory is used to systematically structure the framework and guide practice development, with conceptual ideas consistently applied to support person-centred care and recovery in child and adolescent mental health.*	Nonaka's as its theoretical framework (47)	The SECI model and the concept of Ba can enhance person-centred care in child and adolescent mental health by supporting shared knowledge creation and relational connectedness. They align with recovery principles by emphasizing individual needs, values, and social support. They promote developmentally appropriate, personalized care through emotional connection, collaboration, and mutual understanding between young people, caregivers, and professionals. The approach empowers youth as active participants in their recovery, strengthening resilience through meaningful engagement and shared decision-making. A sense of safety and connection fosters hope, acceptance, and control, which are crucial to supporting recovery in child and adolescent mental health.

CCC, child-centred care; FCC, family-centred care; HCP, healthcare professional; YP, young people.

**Table 2:** Thematic synthesis across theories.

Theme	Bronfenbrenner’s ecological model	Vygotsky’s ZPD	Resilience theory	The child’s perspective new sociology of childhood	Theory of trust	SECI model and the concept of “Ba”	Shared insights	Core domain (Step 3)
Child's Role	Child actively shapes and is shaped by the environment	Child is an active learner who co-constructs knowledge	Child adapts to risk using personal and external strengths	Child is a social actor with views, experiences, and agency	Trust depends partly on the child's interpretation of others’ motives	Children are active participants; their opinions and preferences are integral to the decision-making	Children are **meaning-makers and active agents**	The Competent Child: Agency, Voice, and Meaning-Making
Social Context	Development unfolds in interconnected systems (e.g., home, school, society)	Learning is situated in cultural-historical context	Social ecology of support and adversity affects resilience	Children exist within and respond to sociocultural contexts	Social context influences how trust is built and maintained.	Social relationships, including family, peers, and community are fundamental to the development and well-being of children	Development is **situated in real-life social contexts**	Social Context and Ecologies
Relationships	Focus on interactions within the child's immediate systems	Key learning occurs in interaction with adults or peers	Strong relationships provide protective support	Emphasizes mutual respect and recognition in relationships	Trusting relationships are built through consistent, empathetic interaction	Focus on forming compassionate and supportive relationships with children, focusing on their individual needs, values, and beliefs.	**High-quality, dependable relationships** are crucial	
Developmental Focus	Holistic development over the lifespan	Cognitive and social learning processes	Adaptive development in the face of adversity	Development through meaning-making and participation	Trust as a dynamic psychological and relational process	Holistic development, addressing physical, emotional, social, and cognitive needs.	Development is shaped by **interaction, meaning, and connection**	Development as a Dynamic Process.
Educational/Practice Implications	Promote continuity across home, school, policy	Use play-based approaches to scaffold learning through guided interaction	Build resilience through problem-solving and emotional regulation	Involve children's voices in care and learning	Use play and age-appropriate tools to build trust, ease anxiety, and involve children in their care.	Create supportive environments that foster recovery and rehabilitation	All inform **relational, strength-based, participatory practice, grounded in respect, collaboration and playfulness**	Supportive actions
Support Mechanisms	Support is embedded across systems and over time	Scaffolding enables children to work beyond current abilities	Internal (e.g., self-efficacy) and external (e.g., care) supports foster resilience	Respectful dialogue and participation foster autonomy	Trust grows through consistent, empathetic, and child-centred communication	Knowledge sharing and collaboration	Effective support is **relational, collaborative and built on consistent and respectful engagement.**	

## 4 Step 1: theories applied in child-centred care studies

The included studies (*n* = 13) are grouped under the main theoretical perspective and discussed in the following sections.

### 4.1 Bronfenbrenner's ecological model

Six studies applied Bronfenbrenner's ecological model to frame CCC (21–24, 27, 28). The model conceptualizes development as being shaped by dynamic interactions between a child's individual characteristics (e.g., age, gender, intelligence) and multiple layers of environmental influence (23, 34). Often illustrated as five nested systems––microsystem, mesosystem, exosystem, macrosystem, and chronosystem––the model places the child at the centre, emphasizing how immediate environments (microsystems) exert the most direct influence on psychosocial and emotional development. These systems encompass the child's close relationships, interactions across those settings, broader societal structures, and the dimension of time (28, 34).

Ford, Dickinson (28) argue that Bronfenbrenner's ecological model and more recent bio-developmental models (35) offer a strong theoretical foundation for CCC. The ecological perspective highlights how children's health and development are shaped by their social and physical environments, including relationships beyond the immediate family, such as those with HCPs (28, 36). Several studies stressed the role of trust, collaboration, and child-friendly environments in fostering agency, emotional security, and positive experiences (22, 23, 28) Clarke (22) found that listening to children, providing choices, and maintaining parental presence supported adaptation to hospitalization. Foster, Quaye (23) and Ford, Dickinson (28) further argued that focusing on the child's ecological context; connections, cultural values, and relationships, aligns CCC with the model's principles.

Stålberg, Sandberg (27) applied concepts that position children as competent social actors (37, 38), demonstrating how participation occurs through reciprocal interactions supported by an interactive communication tool. This approach made children's perspectives visible, reinforced agency, and revealed potential to reduce distress and anxiety (27). Similarly, Nicholl, Evelegh (24) incorporated the model into the Respectful Approach to Child-centred Healthcare (ReACH), which operationalizes child-centred principles into trust, authenticity, observation, and environment, showing positive effects on adherence and care experience. At a broader level, Brigden, Shaw (21) applied a socio-ecological perspective to chronic illness care, emphasizing interconnected systems beyond the microsystem. They identified the need to engage schools, share information across settings, and target systemic barriers to integrated care. Collectively, these applications illustrate how the ecological model provides a holistic framework for translating philosophical commitments to children's agency into practical, relational, and context-sensitive strategies.

#### 4.1.1 The ecological model and the united nations convention on the rights of the child (UNCRC)

Seven studies explicitly linked CCC to the United Nations Convention on the Rights of the Child (UNCRC) (9, 22–24, 27, 28, 30). These studies portrayed CCC as requiring an explicit commitment to children's rights in healthcare (9, 24). Foster, Quaye (23) demonstrated how shared decision-making among children, parents, and professionals supports participation aligned with the UNCRC. Similarly, Ford, Dickinson (28) argued that Bronfenbrenner's ecological model complements the UNCRC by showing how children's rights depend on support across environmental systems. For example, informed parents (microsystem) and responsive health professionals (exosystem) play key roles in helping children navigate healthcare, while a society that values children's agency (macrosystem) ensures their voices are respected. Stålberg, Sandberg (27) also emphasized children's agency and the importance of listening to all forms of expression, reinforcing the UNCRC's call to consider children's perspectives a meaningful part of care.

### 4.2 The child's perspective and the new sociology of childhood

Coyne, Hallström (9) and Söderbäck, Coyne (30) applied the New Sociology of Childhood (39) and Sommer, Samuelsson (40) concept of the Child's Perspective to distinguish CCC from family-centred care and to emphasize the importance of children's own viewpoints. Both perspectives reject the view of children as passive recipients, framing them instead as capable, active agents who shape their lives and influence others (9, 39), while recognizing their embeddedness in interdependent family systems (9). The Child's Perspective reflects the child's unique experiences, understandings, and perceived needs (40), requiring professionals to treat each child as a subject with specific competencies rather than making generalized assumptions (9, 30). Implementing these perspectives involves guided participation, mutual engagement strategies such as modelling, questioning, and expanding on the child's contributions, all to support the child within their zone of proximal competence (9, 40). In practice, this means recognizing children as active participants who shape care through interactions with parents and professionals as well as creating opportunities for meaningful involvement. Coyne, Hallström (9) further linked trust, respect, autonomy, and self-determination to CCC, noting how these values influence communication and the regulation of information in healthcare encounters.

### 4.3 Theory of proximal development

DeCosta, Grabowski (25) applied Vygotsky's (41) Zone of Proximal Development (ZPD), along with the concept of scaffolding (42), to identify developmentally appropriate CCC practices. The ZPD represents the gap between what a child can do independently and what they can achieve with support, with learning occurring through social interaction. Scaffolding (supportive tools and actions) enables the child to operate within this zone (41, 42). DeCosta, Grabowski (25) demonstrated that playful communication, humour, and positive feedback were key scaffolding strategies for fostering trust, participation, and engagement. Aligning care with children's everyday experiences helped them express feelings and needs, whereas activities outside the ZPD (e.g., focusing on screens) hindered participation. These findings echo the guided participation concepts discussed by Coyne, Hallström (9) and Stålberg, Sandberg (27), which also emphasize how mutual engagement and shared meaning-making are fundamental to CCC.

### 4.4 Resilience theory

Two studies applied resilience theory to identify strategies that support CCC (31, 33). Both conceptualized resilience as a dynamic process shaped by interactions between individual, social, and environmental factors, where successful adaptation strengthens coping resources and promotes positive development (27, 33, 43–45). Noeker and Petermann (33) conceptualized resilience as a dynamic process shaped by ongoing interactions between children and their environments, highlighting child-centred interventions that support coping and adaptation. Their model emphasizes facilitating successful coping experiences to build self-efficacy and adaptive behaviours through problem-solving, emotional regulation, and positive feedback, enabling children to develop functional cognitive schemas and coping strategies within supportive contexts (33). DeCosta, Grabowski (31) argued that, after a diagnosis of type 1 diabetes, child-centred support functions as a protective factor for psychosocial adjustment by fostering resilience (43, 44). Key strategies included fostering caring relationships and trust, maintaining normalcy and identity, and creating opportunities for meaningful participation through age-appropriate practices and scaffolding. Supportive environments, play-based engagement, and positive feedback further enhanced social competence and resilience (31).

### 4.5 Trust theory

Seven studies identified trust as fundamental to CCC and children's healthcare experiences (9, 22, 24, 26, 28, 31, 32). Clarke (22) found that prolonged hospitalization enabled children to incorporate nurses into their microsystem through relationships grounded in trust. Ford, Dickinson (28) stressed that prior experiences and expectations, such as trust in others, shape developmental processes. Coyne, Hallström (9) identified trust, along with respect and autonomy, as central to child-professional relationships. Nicholl, Evelegh (24) noted that trust underpins participation and grows through honest communication, consistent presence, and respect for autonomy. DeCosta, Grabowski (31) showed that trust in HCPs was crucial to positive hospital experiences, while Attard, Elliot (32) argued that shared experiences create safety and support recovery. DeCosta, Skinner (26) applied Möllering's (46) theory of trust to explore how trust shapes young children's healthcare experiences. Möllering conceptualises trust as a dynamic mental process comprising interpretation (based on past experience), suspension (a leap of faith), and expectation (the resulting state of trust or distrust). This cyclical process means that each interaction shapes future trust. DeCosta, Skinner (26) found that young children, who lack generalized trust and rationalization skills, built trust through repeated, meaningful interactions in which HCPs engaged them on their terms, using play, body language, and imagination to create safety. By entering the child's lifeworld and forming authentic connections, professionals fostered trust, cooperation, and emotional security. When children did not fully trust HCPs, children often relied on their caregivers' trust to accept treatment (26).

### 4.6 The SECI model and the concept of Ba

Attard, Elliot (32) applied the SECI model and the concept of Ba to enhance child- and adolescent-focused mental healthcare. The SECI model (47, 48) describes knowledge creation through interactions between tacit (personal, emotional) and explicit (documented) knowledge via four modes: socialization, externalization, combination, and internalization. This framework supports development of individualized, experience-informed care. Ba refers to a shared physical, mental, or virtual space where relationships and knowledge emerge through shared values and emotional connection (32, 47). Emphasizing dialogue, empathy, and mutual understanding, Ba fosters empowerment, safety, and resilience. Attard, Elliot (32) argued that these concepts adapt adult-oriented recovery models to youth needs, guiding interventions shaped by young people's experiences and caregiver input (32, 49, 50).

## 5 Step 2: towards a coherent framework—a thematic analysis across theories

In Step 2, we conducted a thematic synthesis across the six theories to examine shared perspectives, convergence and conceptual alignments. The purpose was to identify the theoretically grounded elements that underpin CCC (see Table 2).

Across the six theoretical perspectives, several consistent patterns emerged. All theories conceptualised children as active participants who contribute to their own care and development. Likewise, each theory recognised that children's experiences are embedded within social relationships and broader ecological contexts, highlighting the importance of families, healthcare professionals, and supportive environments. Finally, all theories viewed development as an ongoing and relational process, while proposing different mechanisms through which participation, learning, trust, resilience, or knowledge sharing support children's growth and well-being. Together, these shared themes formed the basis for the conceptual refinement undertaken in Step 3.

## 6 Step 3: integration of themes and exposition of relationships

In Step 3, we reviewed the thematic synthesis and conducted a synthesis refinement in which the aim was to generate a conceptual advancement. This step allowed us to move beyond individual theories and highlight how they collectively inform a coherent understanding of CCC, emphasizing the underlying philosophical foundations, theoretical foundation, as well as practical guidance for implementation. The refinement process was iterative and interpretive. Rather than reflecting any single theory, the domains were generated by identifying recurring concepts that were consistently represented across multiple theoretical perspectives. The final domains therefore represent points of theoretical convergence rather than independent theoretical constructs.

From the matrix (Table 2), Social Context and Relationships were collapsed into the domain, Social Context and Ecologies. Support Mechanisms and Educational/Practice Implications were combined into a single category: Applied Support, describing the practical enactment of the theoretical principles. In contrast, The Child's Role and Developmental Focus each remained as distinct, theoretically anchored domains of CCC.

The theories were not treated as contributing equal explanatory weight. Rather, they were synthesised according to the role they played in explaining CCC. Bronfenbrenner's ecological model provided the overarching systems perspective, the Child's Perspective and New Sociology of Childhood offered the philosophical grounding, while the remaining theories contributed complementary explanatory mechanisms relating to participation, trust, resilience, development and knowledge creation. The final framework therefore reflects an integrative synthesis of complementary theoretical contributions rather than a simple aggregation of theories.

This resulted in the identification of three core, interdependent domains of CCC: (1) The Competent Child: Agency, Voice, and Meaning-Making; (2) Social Context and Ecologies; and (3) Development as a Dynamic Process.

**Figure 2:**
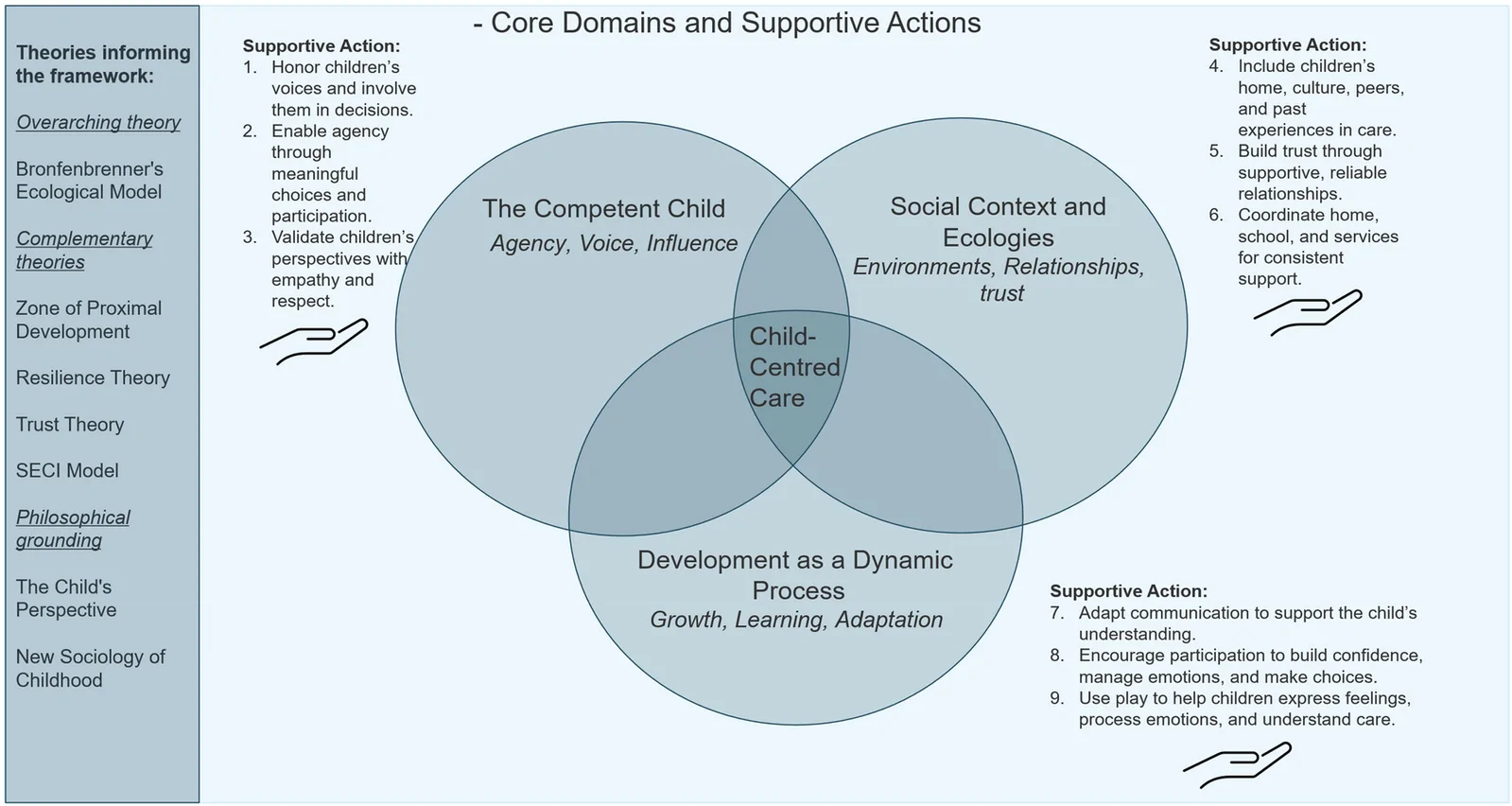
Framework of child-centred care.

These domains emerged consistently across the theories and, through their synthesis, collectively provide a theoretically grounded framework for understanding CCC. The domains are interlinked: For example, recognizing a child as a competent actor requires considering the social environments in which they are embedded, as their capacity to participate is shaped by the support and responsiveness of their family, healthcare providers, and the wider sociocultural context. Similarly, participation is a developmental process that evolves through relationships, learning opportunities, and emotionally safe environments. The domains offer a comprehensive lens through which CCC can be conceptualized and operationalized. The Framework is illustrated in Figure 2, and each domain and its theoretical underpinning are described below.

### 6.1 The competent child: agency, voice, and meaning-making

Central to all identified frameworks is that they recognize the child as a competent, active agent in their own care and development. Bronfenbrenner and Vygotsky present the child as an active participant in shaping their environment and co-constructing knowledge (41, 51). The Child's Perspective and the SECI model further underline children's voices, choices, and interpretations as vital components of decision-making processes (32, 40).

The Theory of Trust underlines how care relationships are co-created through the child's interpretation of others' intentions, highlighting the importance of honouring children's perspectives and fostering their autonomy (26, 32, 40, 46). Resilience Theory and the New Sociology of Childhood support viewing children as resourceful actors capable of harnessing strengths to navigate challenges (33, 39, 44). **In summary, CCC entails respecting and incorporating children's rights, agency and expertise into all aspects of care.**

### 6.2 Social context and ecologies

At the heart of CCC lies an understanding that children develop within complex social ecologies. Bronfenbrenner's ecological model situates the child in interconnected environments, from family and school to broader society; environments that must be considered in care planning (51). Vygotsky and the SECI model emphasize that learning and growth emerge through culturally situated, relational interactions (32, 41). Building trusting relationships are fundamental. The Theory of Trust and the Child's Perspective stress that care is effective only when it is grounded in empathy, respect, and consistent relational engagement (9, 26, 30, 39, 40). Resilience Theory highlights the protective power of supportive relationships in the face of adversity (31, 33, 43, 44). Together, the theories demonstrate how CCC must be **relationally anchored, culturally sensitive, and embedded in real-life social contexts.**

### 6.3 Development as a dynamic process

The reviewed theories collectively underscore the notion that development is complex, adaptive, and multidimensional, necessitating care approaches that are flexible and holistic. The domain emphasises that child-centred care should evolve alongside children's changing developmental capacities, needs, and preferences throughout childhood and adolescence, while recognising that development is shaped through ongoing interactions with others and the surrounding environment. Bronfenbrenner's lifespan systems approach (51), Vygotsky's focus on cognitive and social development (41), and Resilience Theory's emphasis on adaptation under stress frame development as a dynamic interplay between the child and the environment (31, 33, 43, 44). The Theory of Trust adds a relational and psychological dimension, viewing trust-building as an ongoing, evolving process that is critical to effective care (26, 46). The SECI model's focus on knowledge sharing and co-creation within relational spaces further enriches this view (32). CCC, therefore, requires **responsive strategies that address emotional, social, cognitive, and cultural dimensions, supporting children's development as an interconnected, ongoing process.**

### 6.4 Supportive actions: relational and participatory strategies for effective child-centred care

(SupportMechanisms+Educational/PracticeImplications)

Bridging theory and practice, the theoretical frameworks provide guiding principles for delivering CCC. Bronfenbrenner called for alignment across systems to ensure consistent support (51), while Vygotsky advocated learning through participation and guided, playful interaction (41). Resilience Theory highlights fostering emotional regulation and problem-solving capacities (33, 44), and the Child's Perspective insists on active involvement of children in their care decisions (30, 40). The Theory of Trust emphasizes empathetic communication and the creation of safe, trusting environments, often facilitated by play and developmentally appropriate communication tools (26, 46). SECI's collaborative knowledge-sharing model complements these insights by encouraging environments that nurture recovery and empowerment (32). Together, these perspectives reinforce **the notion that CCC must be relational, strength-based, participatory, and tailored to individual needs and contexts.**

To help translate these theoretical insights into real-life practice, we have operationalised nine tangible, practice-oriented strategies based on the three core, interdependent domains. Collectively, these strategies operationalise the three domains by translating the theoretical principles identified across the six perspectives into practical approaches for child-centred care. Strategies linked to *the Competent Child* focus on recognising children's agency, listening to their perspectives, and supporting meaningful participation. *Strategies within Social Context and Ecologies* emphasise trusting relationships, collaboration with families, and responsiveness to children's everyday environments. Finally, strategies associated with *Development as a Dynamic Process* focus on adapting care to children's developmental stages through scaffolded communication, play-based engagement, and opportunities for progressive participation and learning. Together, these strategies demonstrate how the theoretical framework can inform practical, developmentally responsive child-centred care. They are illustrated in Figure 2, aligned with the three domains of CCC.

The strategies are further exemplified in the Educational and Practice Implications section, where practical examples demonstrate how the framework may be operationalised across different clinical contexts and developmental stages.

## 7 Discussion

The present scoping review set out to explore how theoretical perspectives have been used to conceptualize and operationalize CCC within healthcare settings. Specifically, it aimed to identify the theories employed in CCC research, examine the fundamental elements that define the approach, and explore the theoretical insights these perspectives offer for understanding and advancing care for children and young people. The following discussion explores the theoretical foundations and their contribution to CCC and the core components of CCC, which together make up the proposed theoretical Framework of Child-Centred Care, and considers their implications for practice, policy, and future research.

### 7.1 Theoretical foundations and their contribution to child-centred care

A robust theoretical foundation is essential for identifying and understanding the core elements of CCC. Such a foundation enables practitioners and researchers to evaluate not only the effectiveness of these approaches but also the processes through which they achieve impact, providing a basis for refining interventions, guiding implementation, and promoting evidence-based care tailored to children (52). Previous research has indicated that it is often unclear, in the literature, how the key concepts of CCC should be authentically enacted (10). Others have shown that the central concept of participation is often insufficiently grounded in theory (4). Based on our analysis, we propose a theoretically anchored framework that illustrates how the principles of agency, participation, decision-making, and communication must be integrated across three core components—The Competent Child, Social Context and Ecologies, and Development as a Dynamic Process—to be genuinely child-centred. This framework also identifies the essential domains that must be preserved to ensure fidelity across diverse settings, while allowing flexibility for contextual adaptation.

For example, active participation is *required* to acknowledge children as competent actors. It is *essential* for understanding their everyday experiences in context to include these perspectives on care and decision-making. Lastly, participation is *necessary* for development, fostering autonomy, growth, and self-efficacy. Our theoretical framework also illustrates how participation must be tailored to each child's abilities, take place within their ZPD, and often use playful or interactive communication. Likewise, meaningful decision-making must actively support and develops children's decision-making skills and capacity. The process should be collaborative and respect the child's voice, thus promoting their sense of control and empowerment within their care.

Theory serves as a critical tool for understanding the mechanisms through which child-centred practices are expected to influence outcomes: By meaningfully including the child in their care, scaffolding learning and coping strategies, and using developmentally appropriate communication, practitioners help build trust, strengthen relationships, and foster emotional resilience, all of which support the child's overall development in a positive way (44, 53). Exclusion from participation, on the other hand, can result in missed opportunities for children to develop important skills such as communication, decision-making, emotional regulation, and self-advocacy. These are not incidental benefits; they are central to cultivating autonomy, resilience, and a sense of self-efficacy, qualities that are essential to navigating health and illness both in childhood and beyond (54). The theories identified in the present review are all well-established and widely supported across disciplines, and discussing each individually is beyond the scope of the paper. What is more relevant is how their shared insights inform CCC. Consequently, rather than addressing the included theories independently, the following section examines their contributions and discusses how they collectively underpin the three interrelated domains of our proposed Framework of Child-Centred Care.

### 7.2 Core components of child-centred care—a framework of child-centred care

The present review identified three core, theoretically anchored domains of CCC: The Competent Child, Social Context and Ecologies, and Development as a Dynamic Process. The domains are deeply intertwined and mutually reinforcing, shaping both the understanding and practice of CCC. Together, they inform a Framework of Child-Centred Care in which no single domain can stand alone. Because these three domains continuously influence one another, separating them into discrete categories risks oversimplifying their synergistic significance. Nevertheless, for the purposes of clarity, they are, to some extent, discussed individually.

### 7.3 The competent child. Children are competent actors and meaning-makers

One central concept of CCC, identified in the present review, is the recognition that children are competent, active participants in their own care and development. Our review found that, while children are embedded within family systems and significantly influenced by their parents, particularly in early childhood, they are also autonomous individuals who actively interpret, respond to, and shape their environments (41, 55). Several studies included in this review explicitly link CCC, and the recognition of children as competent actors, to a commitment to children's rights in healthcare. The United Nations Convention on the Rights of the Child (UNCRC), particularly Article 12, affirms the child's right to express their views freely in all matters affecting them, while Article 24 recognises the child's right to the highest attainable standard of health and access to healthcare services. Together, these articles provide a rights-based foundation for CCC by reinforcing both participation and access to appropriate, responsive care. Importantly, the theoretical perspectives identified in this review help to elucidate how these rights may be meaningfully implemented in practice.

The recognition of children as competent, active participants aligns with a substantial body of research demonstrating children's desire for active involvement in their care (4). Studies have consistently shown that children value honest, age-appropriate communication and dislike being excluded from conversations or spoken over (3). Even young children wish to be seen as competent individuals, seeking information and opportunities for active involvement (5, 6). Despite this, many children report not feeling included in medical consultations, highlighting a gap between their desire to participate and how care is typically delivered (4, 56, 57). A recent review found that child participation in healthcare has remained limited over the past 50 years (56), despite growing recognition of children as active agents and increasing emphasis on their right to be included in matters concerning them (7). Similarly, another recent review concluded that, although children's participation in healthcare is essential, it remains underdeveloped (4). Their right to participate is often not explicitly recognized or meaningfully implemented by paediatric healthcare teams, and the extent of children's involvement in care still depends largely on the attitudes and practices of individual healthcare providers (4). The review also showed that the absence of theoretical frameworks in studies on children's participation weakens both conceptual clarity and practical implementation, which can result in tokenistic involvement rather than meaningful engagement. The authors argue that the absence of theory limits the ability to connect participation with broader ethical principles like autonomy, agency, and justice (4). These findings underscore the aim of the present review and highlight the importance of grounding concepts such as participation in theory, not only to affirm children's competence, but also to account for the social, ecological, and developmental contexts that shape their experiences, something that is explored in the following section.

### 7.4 Social context and ecologies. Children are embedded in, and influenced by, interconnected (social) systems

At the heart of the theoretical insights explored in the present review is a shared emphasis on the relational and ecological nature of children's development. This highlights the need for care approaches that are flexible, responsive, and embedded in children's everyday experiences (27, 55). As development is ongoing and shaped by children's interactions with caregivers, professionals, and their broader environment, care must also account for the emotional and practical needs of caregivers, especially during hospitalization or management of chronic illness (58). HCPs, too, must be adequately supported, thus provided the time, training, and resources needed to practice CCC (24). Lastly, schools, national guidelines, and policies are all part of connected systems that must work together to support the child (59).

We found that trusting relationships are central within the domain of Social Context and Ecologies and constitute a core element of CCC. This aligns with a broader body of research highlighting the critical role of trust in paediatric healthcare (60), particularly when children face pain, fear, or unfamiliar procedures (61). Pain in children is a complex, dynamic, and subjective phenomenon, shaped by developmental, emotional, and contextual factors (62). It is further influenced by early life experiences as well as the child's sense of trust in the HCP performing the procedure. Even routine interventions can be experienced very differently depending on whether trust has been established (5).

Trust, therefore, is inherently relational and constitutes a primary psychosocial need in paediatric care. It is closely linked to the development of resilience through social support and emotional regulation (54, 63). The ability to manage both positive and negative emotions is central to young children's emotional well-being, shaping their mood and self-esteem (64). This aligns with a recent study showing that trusting relationships with nurses help children feel safe, respected, and valued, while long-term continuity of care fosters deeper trust (3). Conversely, frequent staff changes or inexperienced professionals undermine relationship-building and listening (3).

Building on this, the domain also underscores the importance of engaging with children's everyday experiences and integrating their perspectives into care planning and decision-making. Medical fear often stems from previous experiences of pain, fear, or a sense of lost control (65). Similarly, challenges in treatment adherence can arise from social pressures, emotional difficulties, or unmet needs that routine medical care may overlook (66). These insights indicate that, to be appropriate and meaningful to the child, there is a need for child-centred communication strategies that recognize how children are embedded within and influenced by interconnected social systems. Such strategies are foundational in supporting emotional regulation, resilience, and overall development—topics that will be discussed further in the next section: *Development as a Dynamic Process*.

Lastly, our analysis offers a theoretical contribution to ongoing debates about child-centred vs. family-centred care. Bronfenbrenner's Ecological Model firmly places the child at the centre of their own lifeworld, highlighting their embeddedness in family systems but also their individual agency and experience (55). Particularly in early childhood, family dynamics, and children's strong need for their parents, as emphasized by Bowlby's Attachment Theory, have contributed to the dominance of family-centred care in paediatric settings (67, 68). However, this has sometimes led to an overemphasis on parental involvement at the expense of the child's voice (9, 67). Crucially, CCC does not diminish the role of the family. It recognises the family as one of the most critical systems supporting the child's well-being. Yet it also insists that care should not stop at caregiver involvement (9). Children, regardless of age, are persons in their own right, with unique needs, emotions, and perspectives that must be heard and respected. This position is supported across all included theoretical frameworks and is consistent with philosophical and rights-based approaches to care (3).

As practice shows, family-centred care is often misinterpreted as parent-centred care, sometimes even mother-centred (67). While family support is crucial in helping children understand and express themselves, parental involvement often unintentionally overshadows the child's voice (3). When this happens, caregiver partnership may be mistaken for genuine participation, even when children themselves are excluded from decision-making and interaction (9). As a result, the child's perspective risks being marginalized, contrary to the foundational principles of CCC.

### 7.5 Development as a dynamic process. Children learn, grow, and adapt through participation and supportive relationships

The third core domain of CCC recognizes development as a dynamic, ongoing process that challenges static, one-size-fits-all approaches. Drawing on Bronfenbrenner's Ecological Model, it highlights how children's growth is shaped across emotional, social, cognitive, and cultural domains, within interconnected systems (51). This perspective calls for practice environments that are responsive, relational, and participatory. It points out that no fixed age or developmental threshold is required for meaningful involvement. Rather, when children are engaged within their ZPD, learning and adaptive growth are socially mediated through supportive relationships, which foster resilience and successful coping over time (32, 33, 41, 46).

This understanding is consistent with previous studies that have described how children move from dependence to independence in healthcare through supported opportunities to participate (69, 70). A recent study showed that meaningful engagement depends on developmental opportunities (ZPD), meaning that children's ability to participate grows when they are given space to practice with adult support. These guided moments helped children build confidence, autonomy, and a sense of agency and recognized them as active participants in their care (69).

One practical example of how scaffolding through the ZPD helps children develop emotional regulation is provided by Holaday, Lamontagne (70): A child undergoing repeated medical procedures initially depends on a HCP to manage distress. The adult introduces coping strategies, like positive self-talk or reframing, that help the child regulate emotions. With ongoing support, the child begins to apply these strategies independently. What started as external guidance becomes internalized, marking a shift from dependence to self-regulation. As the child grows more confident, the need for adult scaffolding fades. This progression illustrates the ZPD in action: development occurs through supported participation, leading to increased autonomy and emotional competence (70). Consequently, meaningful inclusion of children in healthcare encounters requires scaffolded or guided participation. Standard adult-style communication is often ineffective in engaging children, and without adaptation, adult-led conversations can inadvertently reinforce power imbalances that marginalize children's perspectives (56, 71, 72).

Our Framework of Child-Centred Care highlights that coping strategies are most effective when they recognize children as competent actors, making the child's own perspective central. Research has shown that children are competent and capable of expressing their needs and managing fear and pain, provided adults listen and adapt their approaches (73), reinforcing the importance of viewing participation across all domains of CCC. Without the child's perspective, adult-led strategies may unintentionally undermine the child's agency and developing coping strategies. A child-centred approach therefore requires participatory care that recognizes and supports children's agency and preferences (73).

The present review found that play-based approaches are essential to engaging young children within the ZPD, ensuring that activities are meaningful and relevant (25). This aligns with a substantial body of research demonstrating that creative and playful approaches are essential to supporting child participation and giving children a voice (74). Play is central to the ZPD, serving as a key way children express emotions, understand information, and communicate (41). According to Vygotsky (41), during play, children act beyond their usual abilities, making it a concentrated space for learning and growth. Existing literature consistently highlights the importance of playful approaches that meet children where they are and create scaffolding for active participation, learning, and development. These approaches include drawing, crafts, dolls or puppets, visual aids, and storytelling through vignettes, picture books, or films (74–77). Visual, play-based and creative tools can create a safe, low-pressure space for interaction without the need for eye contact, and children often see puppets and soft toys as more approachable and equal partners than (unfamiliar) adults (72, 78, 79). Practitioners can normalize concerns by referencing other children's experiences, using stories or examples to enable third-person discussion of sensitive topics and reduce emotional intensity (77).

Play exists firmly within the child's domain, offering a familiar and developmentally appropriate context in which they can engage, express themselves, and shape the direction of interaction (71, 79). Unlike conventional communication, play allows children to participate on their own terms, making it a powerful, yet underutilized, medium for fostering genuine dialogue and agency in care (71, 79–81). The central role of play is also consistent with Article 31 of the United Nations Convention on the Rights of the Child, which recognises the child's right to engage in play and recreational activities appropriate to their age, further reinforcing the importance of play as a fundamental component of child-centred care (82). Evidence for the convincing potential of play is echoed in a recent review on play interventions for paediatric patients, which found that play enhances both the care experience and developmental outcomes across various clinical contexts (83). Play can reduce pain and anxiety during procedures, aid understanding through patient education, complement treatment in recovery and rehabilitation, and help children emotionally adapt to hospital life (83). In this light, we argue that play and creative tools offer a vital, yet often overlooked, pathway for enabling children's participation, supporting their developmental growth (through stress regulation, understanding medical procedures, and expressing their views) and, by extension, that they constitute a fundamental element of CCC.

### 7.6 Strengths and limitations

#### 7.6.1 Strengths

The present synthesis was conducted in three clear steps; it was systematic, rigorous and grounded in the theories drawn from the included studies. The review highlights the important, yet often underutilized, role of theoretical frameworks in informing the foundations of CCC. Each of the theories we found are important in their own right, but we believe greater value has been created in their synthesis. Despite their diverse origins, the theories converge on several key principles: children as active agents, the primacy of trusting relationships, the significance of context, and the need for holistic, participatory, and strength-based care. By synthesizing these perspectives, the review provides a much needed theoretical framework for understanding and implementing CCC across healthcare settings.

#### 7.6.2 Limitations

The review also has several limitations. Although CCC is increasingly acknowledged in paediatric healthcare, few studies have explicitly grounded their work in theory. In many cases, theory functioned more as a background conceptual frame rather than actively informing the research design, analysis and interpretation. Further, the theories identified reflect only those applied in the existing CCC literature and should not be considered exhaustive. Other theoretical perspectives may offer valuable insights and could further inform the development of child-centred approaches. Although our search encompassed all paediatric healthcare settings, the identified literature was concentrated in a limited number of clinical contexts. Consequently, several areas of paediatric healthcare were underrepresented, and future research should explore whether additional theoretical perspectives emerge across these settings to further refine the proposed framework.

Lastly, most studies were conducted in Western healthcare systems, leaving perspectives from non-Western settings underrepresented. As a result, the transferability of current models across diverse cultural and healthcare contexts remains uncertain. Broader application and evaluation of the framework in varied settings are needed to strengthen its global relevance.

### 7.7 Future Directions

Future research should engage more explicitly with theory both in scope and application. First, the conceptual framework developed in this review should be empirically tested and refined in studies conducted with children and families, ensuring that theoretical insights are grounded in lived experiences and diverse healthcare contexts. Second, theoretical frameworks should be more fully integrated across all stages of the research process, rather than serving primarily as conceptual backdrops. Doing so may strengthen the explanatory power of studies and enhance understanding of how CCC operates in practice. Finally, future research should prioritise the application and testing of theory-informed CCC frameworks in non-Western and culturally diverse healthcare settings. Broadening the geographic scope of research is essential to developing a globally relevant and culturally responsive framework for CCC.

#### 7.7.1 Educational and practice implications

The three domains identified in this review, provide a framework for understanding how CCC can be operationalised in clinical practice. Together, they highlight that effective CCC requires approaches that actively support children's participation, respond to developmental needs, and foster trust within the child's everyday context.

For example, when preparing a young child for a medical procedure, healthcare professionals may use play-based approaches, such as demonstrating the procedure on a teddy bear or acting it out using toy figures. By inviting the child to explore the materials, rehearse the procedure, or relate it to their own experiences, the interaction can be situated within the child's zone of proximal development. This not only supports understanding and participation but also helps build trust and strengthens the child- professional relationship, while providing insight into the child's prior experiences and current concerns.

For older children and adolescents, CCC is similarly operationalised through strategies that actively create space for, and scaffold participation. These may include visual communication tools to illustrate and explore treatment options, structured activities to support decision-making. For example, ranking options using colour-coded categories or conversation cards or prompts that allow children to select statements that reflect their experiences and views.

These examples illustrate the need for education and training that embeds CCC as a core professional competency. Healthcare professionals must be supported to view children as active participants in their care and to develop practical skills in listening, adapting communication, and fostering trust-based relationships. Training should include age-appropriate communication strategies and play-based engagement. Interdisciplinary learning is also essential, preparing professionals to work across systems, as emphasized by Bronfenbrenner. In addition, principles of resilience and strength-based care should guide approaches to supporting children's emotional regulation and problem-solving.

In practice, these theoretical insights inform strategies that prioritise participation, autonomy, and relational care. Professionals should actively scaffold children's decision-making by supporting them to understand available options, express preferences, and engage in shared decision-making. Rather than solely explaining procedures, children should be actively involved in making sense of what will happen. In this way, care must adapt to developmental needs using play, guided interaction, and appropriate digital tools. When care is adapted in this way, it supports the creation of safety and trust through responsiveness, empathy, and warmth. Finally, care should extend beyond clinical encounters to integrate family and community contexts, ensuring coherent support across home, school, and healthcare systems, supported by feedback mechanisms that assess children's sense of inclusion and involvement.

## 8 Conclusion

This review addresses a key gap in child-centred research by proposing a theory-driven framework that places the child as an active participant in their lived experience. By integrating the core domains of agency, social context, and development, it makes clear that care cannot be genuinely child-centred unless all three are considered together. In doing so, the framework moves beyond tokenistic approaches to participation and provides a coherent, conceptual foundation for designing and evaluating care that fully recognises children as active participants in their care and their lives.

A central contribution of this review lies in its synthesis of existing theories. By extracting and integrating key elements across diverse theories, we have developed a framework that articulates the essential domains underpinning CCC. Importantly, this framework is intended as a tentative and unifying foundation rather than a definitive or restrictive model. It does not seek to limit theoretical pluralism but instead provides a shared foundation while leaving space for complementary approaches.

The framework offers researchers and practitioners a practical tool to guide, promote, and implement CCC in diverse contexts. While the framework shows promise for research, education, and clinical practice further empirical work is required to test, refine, and extend it. In particular, research conducted with children and families, and across diverse geographic, cultural, and healthcare contexts, is needed to determine its applicability and relevance in practice. Such work will be essential to strengthening the framework's explanatory value and supporting its development as a globally relevant approach to child-centred care.

## Appendix

Database(s): **Ovid MEDLINE(R)**

Search Strategy in MEDLINE (1946 to August 29, 2024):

**Table:**

#	Searches	Results
1	(((adolescen$ or child$ or teen$ or youth$) adj (cent?red or focused)) and (care or health$)).ti,ab,kf,kw.	1,205
2	*Patient-Centered Care/	14,080
3	((patient or person) adj ((cent?red adj (care or nursing or health$)) or (focused adj (care or nursing or health$)))).ti,ab,kf,kw.	18,987
4	or/2–3	29,146
5	exp *Child/	3,977
6	*Adolescent/	5,555
7	(adolescen$ or child$ or teen$ or youth$).ti,ab,kf,kw.	2,041,266
8	or/5–7	2,042,730
9	and/4,8	1,813
10	1 or 9	2,970
